# Superficial Siderosis and Microbleed Restricted in Cortex Might Be Correlated to Atrophy and Cognitive Decline in Sneddon's Syndrome

**DOI:** 10.3389/fneur.2020.01035

**Published:** 2020-09-16

**Authors:** Ming Yao, Jiuliang Zhao, Nan Jiang, Libo Li, Jun Ni

**Affiliations:** ^1^Department of Neurology, Peking Union Medical College Hospital, Peking Union Medical College and Chinese Academy of Medical Sciences, Beijing, China; ^2^Department of Rheumatology, Peking Union Medical College Hospital, Peking Union Medical College and Chinese Academy of Medical Sciences, Beijing, China

**Keywords:** Sneddon's syndrome, cortical superficial siderosis, cerebral microbleed, cognitive impairment, cerebral atrophy

## Abstract

**Objective:** Sneddon's syndrome is a rare non-inflammatory arteriopathy affecting small and medium-sized arteries, characterized by a generalized livedo reticularis and recurrent transient ischemic attack or ischemic stroke. Hemorrhagic stroke was reported in limited cases, but microbleeds and superficial siderosis were rarely issued. We aimed to investigate the hemorrhagic imaging features of Sneddon's syndrome and explore the possible mechanism and clinical relevance.

**Methods:** Clinical data and cerebral MR imaging including T2^*^ sequence of seven consecutive patients with Sneddon's syndrome were reviewed.

**Results:** The most common neurological manifestations were cognitive impairment and stroke attack (71.4%), followed by seizures and movement disorder (28.6%). Cerebral microbleeds were detected in six patients on T2^*^ sequence, all of them presented with cortical microbleeds, only one of them with microbleeds in basal ganglion. More than five microbleeds were observed in four of these six patients. The majority of the microbleeds were predominantly cortical restricted and especially located in the cortical watersheds. Multiple superficial siderosis were identified mainly involving cortical watersheds in five cases. Significant cerebral atrophy with prominent secondary white matter hyperintensities in bilateral cortical watersheds were also observed. Abnormal tortuous and multiple focal occlusion of bilateral distal MCA were shown in one patient by DSA. No stenosis of proximal segment of cerebral arteries was detected in all the patients.

**Conclusions:** This is the first report illustrating abundant cortical microbleeds and superficial siderosis mainly involved the anterior and posterior cortical watersheds in Sneddon's syndrome. The surprisingly identical topographic distribution of hemorrhagic lesions and the obvious atrophy suggest cerebral atrophy might be secondary to the microangiopathy related hemorrhagic lesions and further contribute to the neurological deficit, especially the early cognitive decline in Sneddon syndrome.

## Introduction

Sneddon's syndrome, first reported in 1965 (1), is now considered as a rare neurocutaneous syndrome characterized by an association of a widespread livedo reticularis with stroke. An annual incidence of approximately four patients per million worldwide was estimated with a female predominance. It is a chronic, progressive, arterio-occlusive disease of unknown etiology involving small-to-medium-size arteries, with the pathologic hallmark of endothelia proliferation (1).

Stroke is one of the diagnostic hallmarks of Sneddon's syndrome. The cerebrovascular manifestations are mostly secondary to ischemia in the superficial and deep territories of middle and posterior artery (2–5). Hemorrhagic manifestations are believed to be unusual in Sneddon's syndrome and has been reported in limited cases (2, 4–9), among which only two studies have evaluated cerebral microbleeds (CMBs) (4, 10). Notably, as far as we know chronic superficial siderosis (cSS) has never been reported in Sneddon's syndrome. Llufriu et al. reported a familial Sneddon's syndrome case with prominent microbleeds (10). In a cohort of patients with Sneddon's syndrome, spontaneous hemorrhagic strokes were found in a rate of 7% in all the strokes (4). T2^*^ sequence was available in 22 patients of the whole sample, showing microbleed in four patients (7%) but without further detailed description about the topographic features about CMBs (4). In fact, among the limited cases of cerebral bleeding manifestations, GRE or T2^*^ sequence was applied in only very rare cases (4, 10), which likely leads to an underestimate of hemorrhagic manifestations in patients with Sneddon's syndrome.

In addition to stroke, progressive cognitive impairment can occur in approximately 77% of Sneddon‘s syndrome, which is recently recognized as an important cause of dementia in youth (2, 3). Despite the clinical observation of cognitive decline in Sneddon's syndrome, the underlying mechanisms for cognitive impairment in Sneddon's syndrome remains poorly clarified. Diffuse cortical and subcortical atrophy was common in Sneddon's syndrome (3, 11). Cortical ischemia predominantly seen in the territories of middle cerebral artery and posterior cerebral artery might be responsible for cerebral atrophy mainly in the parieto-occipital region (7, 11). The cumulative effect of recurrent cerebral ischemic episodes including the small cortical infarcts invisible on conventional MRI sequences, the chronic ischemia secondary to diffuse stenosis of the distal branches, and the axonal loss due to interruption of white matter tracts might contribute to cerebral atrophy and vascular cognitive decline in Sneddon syndrome (3, 11–13). However, previous case reports have revealed that cognitive decline or dementia can precede ischemic stroke in Sneddon's syndrome (11, 13–15), even without any chronic infarct lesion observed on MRI (15). These interesting phenomena thus suggest that Sneddon's syndrome related cognitive decline may occur independently of symptomatic ischemic stroke and some other mechanisms rather than ischemia might be responsible for cognitive decline in Sneddon's syndrome.

In the present study, based on data collected in seven consecutive patients with Sneddon's syndrome, we aimed to characterize the hemorrhagic neuro-radiological findings in Sneddon's syndrome and thus to explore the underlying mechanism and clinical relevance.

## Materials and Methods

### Subjects and Clinical Characteristics

Seven consecutive patients with Sneddon's Syndrome diagnosed at our institution were enrolled between 2015 and 2019. Sneddon's syndrome was defined based on the association of a widespread livedo reticularis involving the trunk and/or the buttock and stroke (5). Intensive screenings, such as test for coagulation profile, lupus anticoagulant, antinuclear antibody, antineutrophil cytoplasmic antibodies, antiphospholipid antibodies, antiphospholipid antibodies, cryoblobulin, protein C, protein S, antithrombin, APC resistance, tumor markers, virus antibodies for HIV, syphilis and TORCH, and cerebral spinal fluid analysis, were performed to make sure whether the patient accompanied with antiphospholipid syndrome or systematic lupus erythematosus, and to rule out other acquitted and inherited non-inflammatory vasculopathies which might contribute to livedo reticularis and stroke. Written informed consents were obtained from the patients or their legal surrogates.

Detailed medical records were reviewed. Demographic data, cardiovascular risk factors (hypertension, smoking, diabetes, overweight, dyslipidemia, atrial fibrillation), and revealing clinical manifestation were collected. Neurological data such as cerebral infarcts, transient ischemic attack and hemorrhagic strokes were recorded. Mini-mental state examination (MMSE) was assessed in five of the seven patients, cognitive impairment was recorded as absent or present in the other two patients.

### MRI Data

Cerebral MR imaging data were performed using a 3.0-tesla (Siemens Skyra or GE MR 750) and all images at diagnosis were reviewed by two experienced neurologists (M. Y. and J. N.) blinded to clinical data. Disagreements were made by consensus. All the patients were undertaken the cerebral imaging, including T1WI, T2WI, fluid attenuated inversion recovery (FLAIR) images (slice thickness 5 mm, gap 1 mm), diffusion weighted sequences (DWI) (b 0, 1,000; slice thickness 5 mm), T2^*^(slice thickness 5 mm, gap 1 mm)/ susceptibility weighted imaging (SWI) images (TR 27 ms, TE 20 ms, FA 15°, slice thickness 1.5 mm) and 3D TOF MRA (TR/TE = 18/3.4 ms; flip angle = 20°, slice thickness 1.2 mm).

T2^*^ sequence or SWI was used to assess the presence of cSS and CMB. CMB was defined as rounded hypointense foci≤5 mm in diameter on T2^*^/SWI sequence distinct from vascular flow voids, leptomeningeal hemosiderosis, or nonhemorrhagic subcortical mineralization (16, 17). The number and location (deep or subcortical distribution) were recorded separately. cSS was defined as linear residues of chronic blood products in the superficial layers of the cerebral cortex showing a characteristic “gyriform” pattern of low signal on T2^*^-GRE/SWIimages, without corresponding hyperintense signal on T1-weighted or FLAIR images (18, 19). Cerebral atrophy was assessed with the visual Cardiovascular Health Study scale (20).

## Results

### Demographics and Clinical Symptoms

The general characteristics of the seven patients are summarized in Table 1. There were three women and four men, with a mean (SD) age at diagnosis of SS was 33.14 (15.27) years. All the patients had a history of hypertension. All of the seven patients presented livedo reticularis (**Figures 2A–C**) before the first neurological symptoms. Cognitive impairment (with the lowest MMSE score of 12) and ischemic stroke attack were found in five patients (71.4%), one of them presents cognitive decline without prior ischemic stroke attack. Hemorrhagic stroke was not recorded. Seizures were found in two patients (28.6%), movement symptoms such as chorea and static tremorin two patients (28.6%). Heart valve involvement were found in three patients (42.9%). Antiphospholipid antibodies were found positive in five patients (71.4%), one of whom with a positive anti-dsDNA antibody. No evidence of infection, neoplasm, cryoglobulin and thrombophilia was found.

**Table 1:** Demographic, clinical symptoms, and radiological characteristics of the seven consecutive patients with Sneddon's Syndrome.

**Case**	**Gender**	**Age**	**LR**	**Neurological symptoms**				**Histroy of hypertention**	**Therapy on admission**		**Antiphospholipid antibodies**	**MRI Presentation**				
				**Cognitive decline**	**Ischemic stroke**	**Epilepsy**	**Movement disorders**		**Antiplatelet**	**Anticoagulation**		**Acute ischemic lesion**	**Chronic ischemic lesion**	**CMB**	**cSS**	**Atrophy^#^**
1	F	23	+	–	–	+	–	+	–	–	+	–	+	+^*^	–	4
2	F	63	+	+	+	–	+	+	+	–	+	+	+	+	+	5
3	F	35	+	–	–	+	–	+	–	–	+	+	+	+	+	4
4	M	17	+	+	+	–	–	+	–	–	+	+	+	+^**^	+	8
5	M	40	+	+	+	–	–	+	–	–	–	–	+	+^**^	+	6
6	M	30	+	+	+	–	–	+	+	–	+	–	+	+^**^	+	5
7	M	24	+	+	+	–	+	+	+	–	–	–	+	–	–	2

# *cerebral atrophy was evaluated based on the visual Cardiovascular Health Study scale*.

+* *indicating patients with more than 5 cerebral microbleeds but still numerable*.

+** *indicating patients with innumerable number of cerebral microbleeds. LR indicates livedo reticularis; CMB, cerebral microbleed; cSS, cortical superficial siderosis*.

### MRI

**Figure 1:**
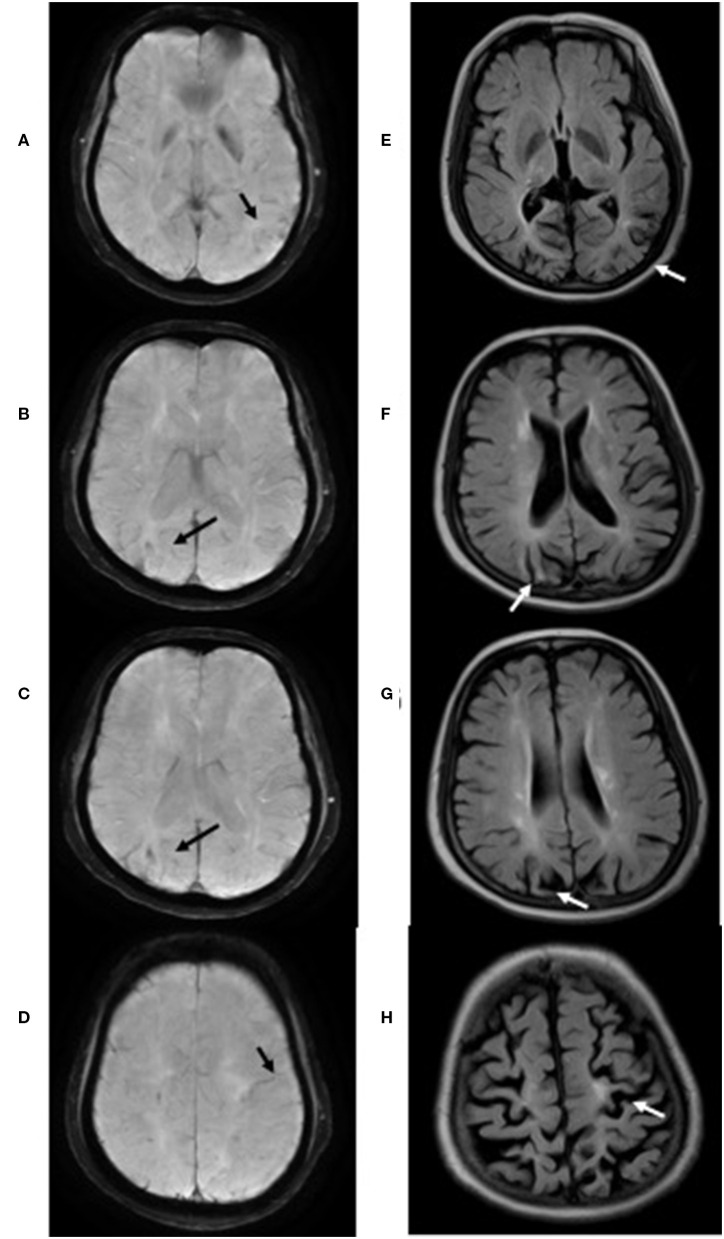
T2^*^ sequences of case 3 showed cortical superficial siderosis (black arrow) mainly involving the left temporal lobe **(A)**, right parieto-occipital region **(B–D)** and left central **(D)**. Significant cerebral atrophy (white arrow) was observed in the regions above-mentioned on Flair sequences **(E–H)**.

**Figure 2:**
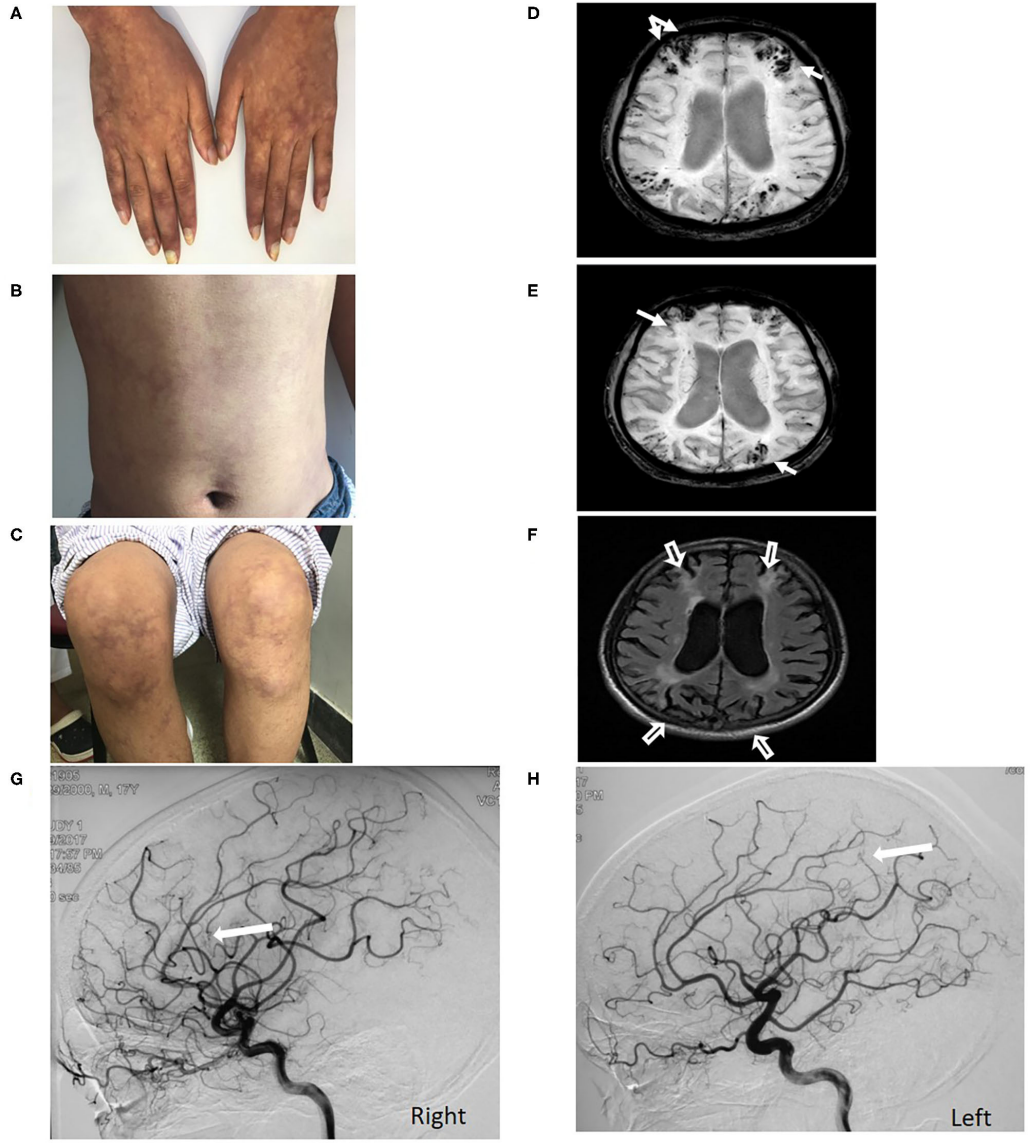
Livedo reticularis of Case 4 over the hands, abdomen and leg **(A–C)**. Multiple microbleeds restricted in cortex and superficial siderosis (white arrow) located in the cortical watersheds are observed on SWI **(D,E)**. Significant cortical atrophy (hollow white arrow) in bilateral cerebral cortical watershed was observed in the context of a generalized cerebral atrophy on Flair sequence **(F)**. DSA shows filling defects (white arrow) in distal segments of bilateral MCA **(G,H)**.

**Figure 3:**
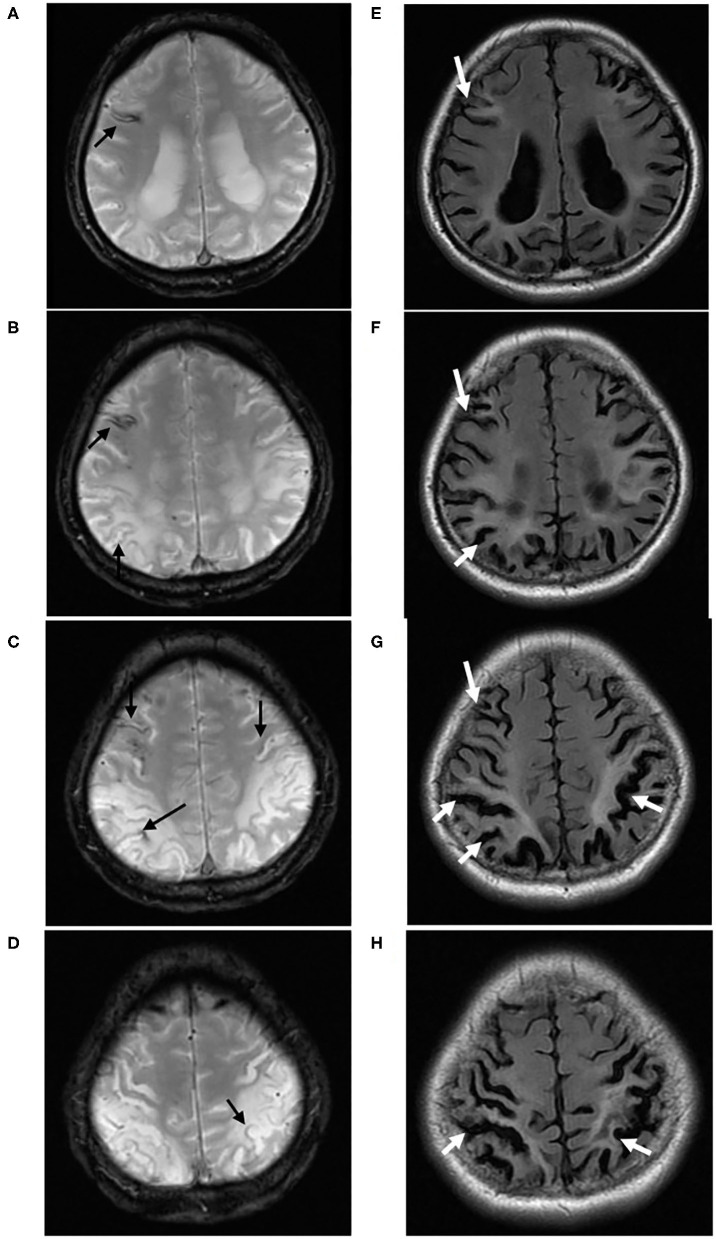
A 40-year-old male (case 5) with Sneddon's syndrome. Multiple chronic superficial siderosis (black arrow) are observed on T2* sequence, predominantly located in the cortical watersheds **(A–D)**. On Flair sequences, prominent white matter hyperintensities and cortical atrophy (white arrow) were found surrounding cSS, especially in bilateral parieto-occipital regions **(E–H)**.

**Figure 4:**
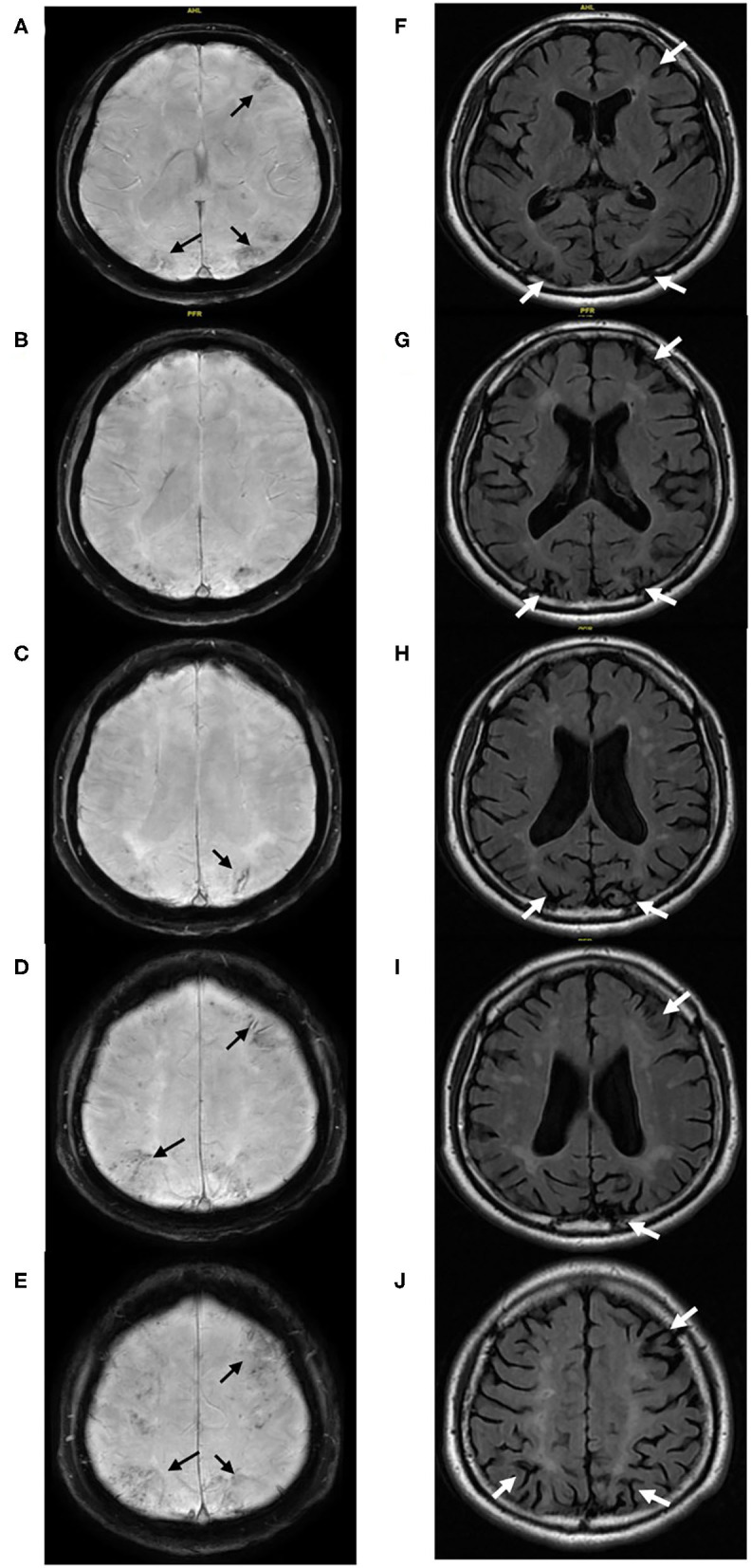
(Case 6) Multiple cerebral microbleeds predominantly restricted in the cortex and chronic superficial siderosis (black arrow) located in the anterior and posterior cortical watersheds are detected on SWI **(A–E)**. The corresponding cerebral atrophy (white arrow) and associated white matter hyperintensities were found on Flair **(F–J)**.

**Figure 5:**
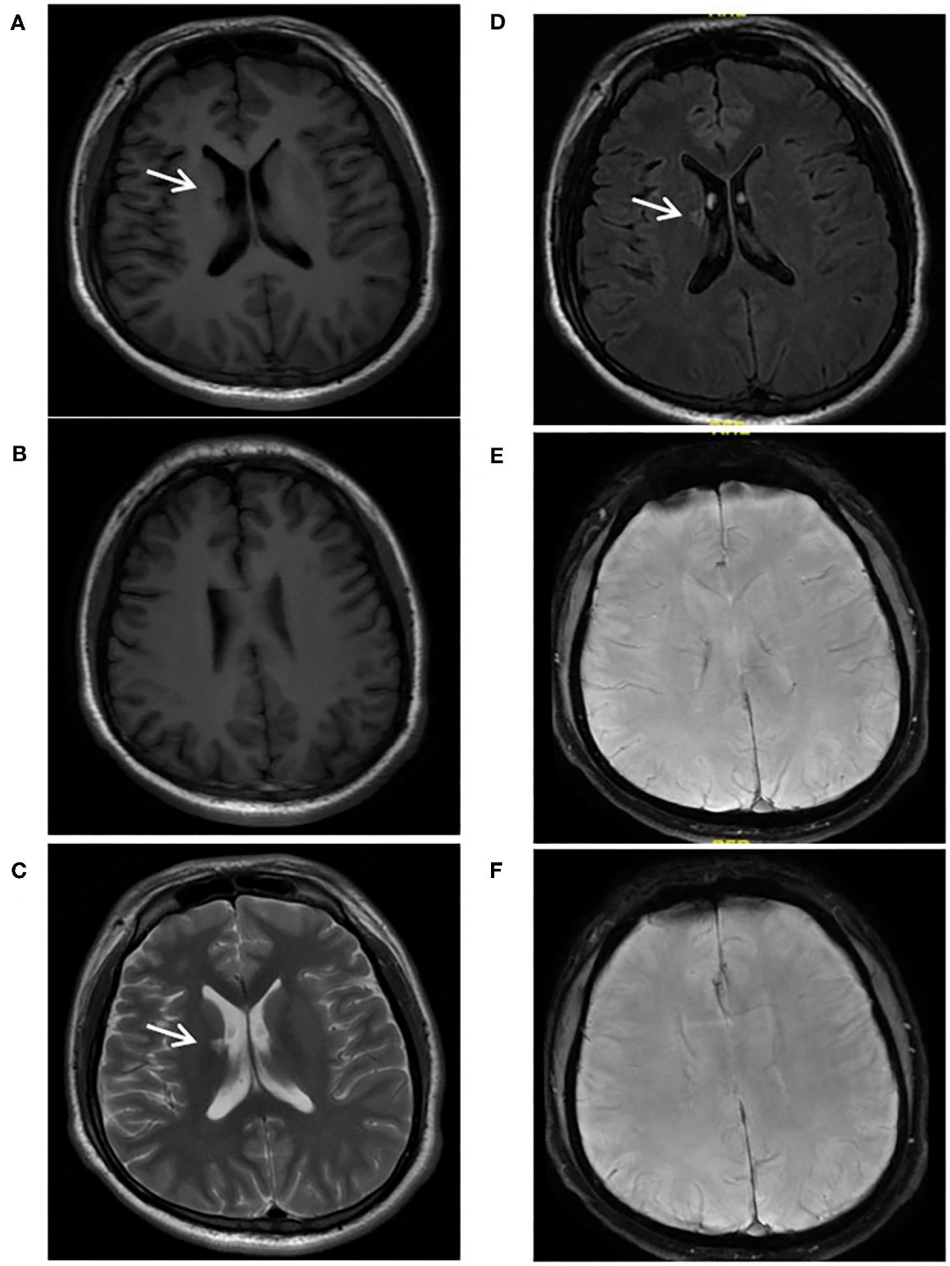
A 24-year-old male with Sneddon's syndrome (case 7) suffered livedo reticularis and ischemic stroke. A chronic lacune was observed in the right basal ganglion **(A,C,D)**. Neither chronic superficial siderosis nor cerebral microbleed was found on SWI **(E,F)**. No obvious atrophy or white mater hyperintensities was found **(A,B)**.

Chronic ischemic lesions were detected in all the patients, mainly involving subcortical areas. Three patients presented with acute ischemic lesions with a characteristic appearance of multiple or single dot-like subcortical DWI high signals involving the temporal, pariental and occipital lobes. Cerebral microbleeds, predominantly restricted in the cortex (**Figures 2**, **4**), were detected in all but one patient on T2-star sequence, four of whom presented more than five microbleeds. CMBs in basal ganglion was only observed in one patient with a number of 3. No CMBs in brain stem was detected. Multiple chronic superficial siderosis (cSS), mainly involving the anterior and posterior cortical border zones, were found in five patients (Figures 1–4). Significant cerebral atrophy in bilateral cortical watersheds, accompanying with prominent secondary white matter hyperintensities, were simultaneously observed, although a diffuse atrophy involved the whole brain. Of note, no obvious cerebral atrophy was observed in the patient without CMB and cSS (Figure 5).

### MRA/DSA

Magnetic resonance angiography of all the patients were normal. Two patients received digital subtraction angiography, one of whom showed abnormal tortuous and multiple focal occlusion of distal segment of bilateral middle cerebral arteries (Figure 2).

### Treatment

None of the seven patients included in the present study underwent anticoagulant agents at admission to our hospital, three of them received antiplatelet therapy previously. Two patients underwent a dual antiplatelet therapy with a combination of aspirin and clopidogrel, while the other one received a mono-antiplatelet therapy with aspirin. After a diagnosis of Sneddon's syndrome in our hospital, warfarin was prescribed for four of five patients with aPL positive, aspirin was recommended to the other one with aPL positive due to a high-risk of hemorrhage. Both of the two patients with aPL negative received aspirin to prevent stroke.

## Discussion

Sneddon's syndrome is a rare non-inflammatory arteriopathy affecting small and medium-sized arteries, characterized by a generalized livedo reticularis and recurrent ischemic stroke. Although hemorrhagic stroke has been described in few cases (1–3), microbleeds and cSS were rarely issued previously (4, 10), which might be underestimated owing to the limitation of radiological technique. To our knowledge, our series is the first report illustrating abundant microbleeds restricted in cortex and cSS involving the anterior and posterior cortical border zones in Sneddon's syndrome. These findings expanded the spectrum of imaging characteristics of Sneddon's syndrome. Moreover, due to the correlation between the location of cSS and CMBs and areas of focal cortical atrophy, the present study might allow for a better understanding of the pathophysiological mechanisms responsible for cognitive decline in this rare syndrome.

The mechanisms underlying the CMBs and cSS in Sneddon's syndrome remain largely unknown. Due to a predominance of CMBs in cortex but not in basal ganglion and brain stem, hypertension appears unlikely to be of importance for the pathogenesis of CMBs in Sneddon's syndrome, although all the patients had a history of hypertension which has been considered as a major risk factor of CMBs (16). The obvious hemorrhagic appearance observed on MRI could not be explained by medication use, because none of the seven patients received anticoagulant agents before admission to our hospital. Cerebral amyloid angiopathy was not taken into consideration due to an early onset of age. Other acquitted and inherited non-inflammatory vasculopathies was ruled out based on the normal or negative results from intensive screening. Abnormal tortuous and multiple focal occlusions of distal segment of bilateral middle cerebral arteries were observed in Case 4 but without evidence of endothelial proliferation in DSA. No brain biopsy was available because of the invasive nature, while the renal biopsy of the same patient illustrated narrowing of small vessel due to prominent proliferation of endothelial cells (data not shown), which is considered as a major pathological hallmark of Sneddon's syndrome (1, 21). In the future, advanced imaging technology may enable the noninvasive visualization of endothelium of cerebral small vessel *in vivo*, and thus contribute a better understanding of the pathogenesis of Sneddon's syndrome. Endothelial activation and dysfunction may play an important role in the pathogenesis of cerebral small vessel disease and contribute to the hemorrhagic manifestations such as CMBs and cSS (22–24). Endothelial dysfunction was found to be independently associated with the presence of CMBs (24).

Superficial branches of the MCA were predominantly involved in Sneddon's syndrome based on the ischemic location (2–5). Abnormal tortuous, irregular stenosis and even obstruction of distal branches with normal proximal MCA were detected in the present case, in line with the findings from previous reports (7, 8, 25), which also showed anastomosis of leptomeningeal and transdural vessels especially in the border zones (7, 25). Taken together, the dilatation and rupture of proliferating and fragile collateral vessels secondary to the stenosis of the distal branch of cerebral arteries might be responsible for the obvious hemorrhagic appearances on T2^*^ or SWI, similar as the mechanisms well described in moyamoya disease (26). Recanalization of the arteries and arterioles within the superficial white matter and leptomeninges and arteriovenous malformations involving meningeal branches have also been observed in Sneddon's syndrome (1, 2), which might also contribute to the development of CMBs and cSS in Sneddon's syndrome.

Of note, significant cerebral atrophy in bilateral cortical watershed areas was simultaneously observed in these patients, in line with previous results (3, 11). As mentioned above, the surprisingly identical topographic distribution of hemorrhagic lesions and the obvious cerebral atrophy made us probably speculate that cerebral atrophy might be secondary to these microangiopathy related hemorrhagic lesions and further largely contribute to the neurological deficit, especially the early cognitive decline in Sneddon's syndrome, although further investigation is needed to elucidate the precise underlying pathogenesis. First, previous reports illustrating patients with cognitive impairment prior to ischemic stroke in Sneddon's syndrome indicate that cognitive dysfunction may not only relate to ischemic progress (11, 13–15). Moreover, progression of hyperintensive signal in T2WI over 6-year follow-up was observed in the majority of patients with Sneddon's syndrome but without obvious deterioration of pre-existent cortical atrophy (3). Second, growing evidence demonstrates that CMBs and cSS are highly prevalent in memory clinic population and in patients with AD (27–31) than those of the general population observed in the Rotterdam Scan study (32, 33). CMBs have been related to impaired cognition in healthy elderly population (33, 34) and in patients with CADASIL or CAA (35, 36). Cognitive decline has also been found to be associated with cSS, especially in patients with disseminated cSS (37–39). Several pathological processes, including microbleeds, cSS and the related pathologies, might affect the cerebral network and thus contribute to cognitive decline in Sneddon's syndrome. Findings from histopathologic studies have illustrated that the presence of CMBs indicates widespread damage in arterioles, resulting in microstructural damage of the surrounding white matter (40, 41), which might account for the white matter hyperintensities observed on MR images. Multiple lobar microbleeds might contribute to cognitive dysfunction due to their direct or indirect effects to surrounding brain tissue invisible on conventional MRI and thus leading to a disconnection of functionally important cortical and subcortical structures involved in cognitive function (42). Evidences from studies on superficial siderosis of the central nervous system, another rare disease with siderosis predominantly beneath the pia on the brain and spinal cord, has showed progressive reduction of cerebral blood flow and oxygen metabolism in the brain stem, cerebellar hemispheres and temporal lobes where often showed marked depositions of hemosiderin and atrophy on MRI. Pathological findings further demonstrated that the presence of hemoglobin along the subpial surface of the central nervous system will result in synthesis of ferritin and hemosiderin, leading to demyelination, proliferation of microglia and neuronal injury in the related areas (43). Finally, only minor cerebral atrophy and slight white matter hyperintensities were found in one of the seven patients, who suffered ischemic attack but presented neither with CMB nor cSS. This interesting finding further supports the above hypothesis.

The present study is the first study that focused on topographic characteristics and clinical significance of hemorrhagic manifestations, including CMB and cSS evaluated on T2^*^ or SWI, in a series of Sneddon's syndrome. Our study has some limitations. First, the sample size is small due to the rarity of this syndrome. Second, because of the cross-sectional observational design of this study, the exact contribution of cSS and CMB to the atrophy was not assessed.

In conclusion, the presents results highlight a better understanding of the typical neurological imaging characteristics of Sneddon syndrome, as well as a better understanding of pathophysiological mechanisms responsible for atrophy and cognitive decline in Sneddon syndrome. Microbleeds restricted in cortex, cSS related to cortical watershed and the secondary atrophy might be highly indicative of Sneddon syndrome in youth with cognitive decline and stroke. Future studies are needed to investigate the pathogenesis and longitudinal progression of cSS.

## Data Availability Statement

The raw data used and/or analyzed during the current study are available from the corresponding author on reasonable request.

## Ethics Statement

The studies involving human participants were reviewed and approved by The Ethics Committee of Peking Union Medical College Hospital. Written informed consent to participate in this study was provided by all the patients or legal guardians.

## Author Contributions

MY contributed to the analysis and interpretation of the data, the study design and the writing of the manuscript. JZ, NJ, and LL contributed to the collection and interpretation of data. JN contributed to the interpretation of the data, the study design and the revision of the manuscript. All authors contributed to the article and approved the submitted version.

## Conflict of Interest

The authors declare that the research was conducted in the absence of any commercial or financial relationships that could be construed as a potential conflict of interest.
